# Rectourinary Fistula after Radical Prostatectomy: Review of the Literature for Incidence, Etiology, and Management

**DOI:** 10.1155/2011/629105

**Published:** 2011-01-26

**Authors:** Hiroshi Kitamura, Taiji Tsukamoto

**Affiliations:** Department of Urology, Sapporo Medical University School of Medicine, South 1 West 16, Chuo-ku, Sapporo 060-8543, Japan

## Abstract

Although rectourinary fistula (RUF) after radical prostatectomy (RP) is rare, it is an important issue impairing the quality of life of patients. If the RUF does not spontaneously close after colostomy, surgical closure should be considered. However, there is no standard approach and no consensus in the literature. A National Center for Biotechnology Information (NVBI) PubMed search for relevant articles published between 1995 and December 2010 was performed using the medical subject headings “radical prostatectomy” and “fistula.” Articles relevant to the treatment of RUF were retained. RUF developed in 0.6% to 9% of patients after RP. Most cases required colostomy, but more than 50% of them needed surgical fistula closure thereafter. The York-Mason technique is the most common approach, and closure using a broad-based flap of rectal mucosa is recommended after excision of the RUF. New techniques using a sealant or glue are developing, but further successful reports are needed.

## 1. Introduction

Radical prostatectomy (RP) is a common treatment for patients with clinically organ-confined prostate cancer. RP is associated with complications such as urinary incontinence, erectile dysfunction, and rectal injury. Since the standardization of the anatomic retropubic RP (RRP), optimization of the surgical technique has been pursued with the purpose of reducing complications [1].

A rectourinary fistula (RUF) is an abnormal opening between the rectum and the bladder or the urethra that results in fecaluria, pneumaturia, and drainage of urine per anus. RUF is a rare but major complication of RP [2]. Management has been traditionally based on urinary or fecal diversion in the hope of spontaneous closure [3], but most of the patients require surgical closure even after such diversions, which means that urinary or fecal diversions tend to be preparatory maneuvers prior to surgical repair. Although surgical approaches, including perineal, transrectal, transsphincteric, and transanorectal repairs are well known, there is no standardized treatment for RUF because of its low prevalence. In this paper, we focus on the incidence and treatment of RUF after RP, and the minimally invasive and most promising treatments are also highlighted and discussed.

## 2. Incidence and Diagnosis of RUF after RP

Rectal injury during RP is one of the main etiologies of RUF. It can occur during apical dissection while attempting to develop the plane between the rectum and Denonvilliers' fascia [4]. Previous series of community-based practice demonstrated 1.5% to 2.2% incidences of rectal injury during radical retropubic prostatectomy (RRP), and 0.6% to 9% of the cases were finally diagnosed as RUF [5–7]. RUF can appear after RP, even if there is no finding of rectal injury during the operation. In the series of 689 RRP and 59 cystoprostatectomies of Noldus et al. [8], 25 rectal injuries occurred. Although 23 of them were diagnosed intraoperatively and closed, RUF developed in 13 patients thereafter [8]. Thomas et al. [9] reported that a third of patients with RUF experienced rectal injury during RRP, which was closed in 2 layers immediately. RUF mostly develops a few weeks after RP, but the range of days is quite wide [10].

There are four surgical options for removing the prostate, RRP, radical perineal prostatectomy (RPP), laparoscopic RP (LRP), and robot-assisted laparoscopic RP (RALP). Comparative studies of LRP versus RRP [11–17], RALP versus RRP [18–20], and LRP versus RALP [21] demonstrated that the incidences of rectal injury in RRP, LRP, and RALP were 0% to 3%, 0% to 2.8%, and 0% to 0.15%, respectively. The incidences of RUF in RPP were reported to be 1 to 1.5% [9, 22, 23]. No study showed a significant difference in the prevalence of rectal injury for any RP procedure, except for a retrospective one [9], which demonstrated that the risk of RUF was 3.06-fold higher for RPP versus RRP. Thus, RUF can occur after any RP technique.

Diagnosis of RUF is not difficult. The clinical presentation of RUF depends on the size of the fistula. Patients usually complain of fecaluria and/or pneumaturia and also watery stool. Fecaluria seems to be a poor prognostic sign [9]. Whatever the clinical symptoms, retrograde urethrocystography, urethrocystoscopy, and rectoscopy or colonoscopy are essential to determine the best management strategy [10, 24].

## 3. Impact of Prior Prostate Radiation on RUF after RP

Radiotherapy-induced cystitis, fibrosis, and tissue plane obliteration are factors that can lead to rectal injuries [25]. A retrospective study from Cleveland Clinic [26] reviewed 22 patients with prostate cancer who were managed with radiation-induced RUF. Six patients were treated with brachytherapy (BT) alone, 5 with external beam radiation therapy (EBRT) alone, 10 with BT + EBRT, and 1 with RP + salvage EBRT [26]. Successful RUF closure was achieved in 9 patients, but 4 of them underwent proctectomy [26]. In a series between 1977 and 2002 from the Mayo Clinic, RUF after EBRT, BT, and EBRT + BT occurred in 30%, 30%, and 40%, respectively [27]. A prospective study from UCSF reported that 7 of 16 patients with RUF underwent prior RP and the other 9 had been treated with BT, EBRT, or cryotherapy [2]. Thus, RUF can be caused by radiotherapy alone.

Therefore, prostate radiation prior to RP is a risk factor for RUF. The incidence of rectal injury after salvage prostatectomy ranges from 2% to 15% [25]. Gotto et al. [28] reported that RUF developed in 22% of men with rectal injury and in 2% of those without it in the salvage prostatectomy group and in 0% of men with it and 0.06% of those without it in the RP group. There was a significant association between rectal injury and subsequent RUF after salvage prostatectomy but not after RP. Although the population of such patients is unique, the difficulty of management for the subsequent RUF with low success rates at surgical repair should be taken into account.

## 4. Conservative Approach

There are few successful reports of conservative management of RUF, which indicates the therapeutic limitations of this approach. In the series of 1447 RP of Thomas et al. [9], three of 13 patients with RUF were treated conservatively without colostomy or surgical closure. These patients showed no fecaluria, and the fistula closed spontaneously during transurethral catheterization after 28 to 100 days [9].

If the RUF is not closed after 3 months of catheterization, further treatment should be considered. The second step of treatment for RUF is fecal diversion. Colostomy was performed for patients with RUF for initial management in a series from the Mayo Clinic, but all of these patients required definitive surgical intervention because of the lack of spontaneous closure [29]. Thomas et al. [9] reported that 33% of patients who underwent colostomy and insertion of a transurethral catheter displayed spontaneous closure of the RUF 23 to 99 days after colostomy. Thus, fecal diversion does not always result in spontaneous closure. For patients without closure at 3 months after fecal diversion, the next step, surgical closure, is recommended. The timing of surgical closure advocated is 2 to 3 months after colostomy [9, 30], since the tissue should be allowed to restore itself for 2 to 3 months prior to fistula repair [31]. If the surgical closure provides a promising result like a very low anterior resection for rectal cancer, loop ileostomy can also be considered for fecal diversion. For the treatment of RUF, however, colostomy seems to be preferred, since it is still a challenging procedure. Thus there has been only one report [32] in which ileostomy was performed with Soave's procedure.

## 5. Surgical Treatment Methods

### 5.1. Approach

**Figure 1:**
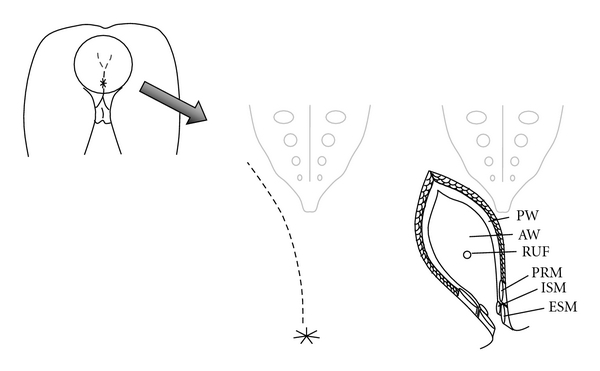
The York-Mason technique. PW: posterior wall of the rectum; AW: anterior wall of the rectum; RUF: rectourinary fistula; PRM: puborectal muscle; ISM: internal sphincter muscle; ESM: external sphincter muscle.

Various approaches, including transperineal, transanal, posterior pararectal, transabdominal and transvesical, transsphincteric, and combined ones [10], have been reported for RUF. If an omental or gluteal muscle flap is planned for closure of the RUF, the transperineal approach is mandatory. However, this approach should be considered only when the fistula is located between the rectum and urethra anterior to the prostate (recto- “urethral” fistula), because the space is too small to expose and repair RUF superior to the pubic bone. It can also be technically difficult because of scar tissue and has been associated with urinary incontinence and urinary stricture [33]. The transsphincteric (York-Mason) and the transsacral (Kraske) approaches, providing excellent exposure, are preferable and often chosen these days. The procedures are described in Table 1. Since RUF occurs at the low rectum, the York-Mason procedure is sufficient to expose the operative field around the RUF in most cases. Moreover, the morbidity of the Kraske procedure is greater than that for the York-Mason one. From 15% to 25% of patients who undergo the Kraske procedure develop rectocutaneous fistula [34], whereas 5% to 7% do after the York-Mason [24] procedure. Thus the York-Mason approach is considered the most appropriate procedure with minimal morbidity (Figure 1). The largest study using this approach, reported by Renschler and Middleton [35], contributed the majority of knowledge about the management of RUF. In the York-Mason procedure, however, layered closure of the anal sphincter is mandatory not only to avoid the risk of fistula but also to maintain fecal continence [10]. The external and internal sphincters and puborectal muscles should be separately demarcated by stay sutures for better identification during closure.

### 5.2. Technique of RUF Closure

**Figure 2:**
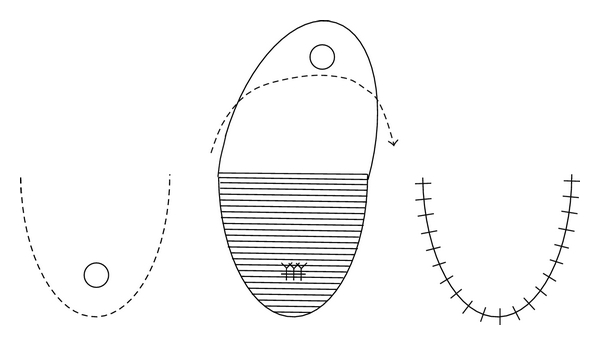
Rectal advancement flap [3].

After exposure of the anterior surface of the rectal wall, the RUF is usually resected with a wide margin. Then the bladder or urethral defect is closed with absorbable interrupted sutures in one layer. If the RUF is in the urethra, the sutures should be performed in a transverse fashion to avoid urethral stricture [10]. There are several procedures for closure of the rectal defect. Although simple layer-to-layer closure seems to be effective, the rectal flap method can prevent recurrence of the RUF at the suture site (Figure 2). Of the rectal flap methods, the Latzko technique may provide the most promising outcome. This procedure was developed for vesicovaginal fistula with a high success rate [59] and applied to RUF by Noldus et al. [8]. To prevent recurrence of RUF, it is important not only to close the fistula in a layer-to-layer fashion but also to eliminate the possibility of contact between the urinary and rectal mucosae. 

A major alternative technique to prevent recurrent RUF is gracilis muscle interposition. The gracilis is the most superficial muscle on the medial side of the thigh, arising from the symphysis pubis and inferior pubic ramus [44]. After a perineal skin incision, dissection at the RUF, and closure of the RUF in 2 layers, the gracilis muscle is harvested, rotated, and placed into the anterior perineal space with fixation about 3 cm above the RUF site [30, 42–45]. This technique provides a high success rate and is one of the most promising treatments for RUF. Recently, Spahn et al. [41] reported 5 patients with RUF who underwent buccal mucosa graft interposition with successful closure, although more cases should be included to validate the result. 

Although there have been few studies of RUF repairs that compared the outcomes of nonradiating fistulas with radiating ones, Vanni et al. [60] recently reported the largest experience of a total of 74 patients. RUF repairs with an anterior perineal approach and muscle interposition flap were performed with success rates of 100% and 84% in nonradiating and radiating cases, respectively. From the results of several studies [26, 27, 60], the muscle interposition flap is considered the most promising method. However, some patients need aggressive treatments such as fecal and urinary diversions with proctectomy [26].

### 5.3. Endoscopic, Laparoscopic, and Robotic Repairs

Minimally invasive approaches, including endoscopic, laparoscopic, and robotic ones, are under development. There were 3 reports [50–52] of transanal endoscopic microsurgery. This technique needs a large endorectal microscope with a suction/irrigation channel and 3 working channels, as well as specially designed scissors, forceps, and needle folders. However, there have been only 5 cases reported in the literature [50–52]. Sotelo et al. performed laparoscopic and robotic repairs of RUF after RP for one patient each [54, 55, 61]. Both patients had a rectovesical fistula, and interposition of the omentum on the rectal sutures was carried out. The laparoscopic and robotic techniques provided successful closure of the RUF. These techniques are feasible, but special devices and technical skills are required.

## 6. New Approaches Using Sealant or Glue

Verriello et al. [62] reported the successful use of a commercial fibrin sealant (Quixil) in combination with an anterior mucosal flap for treatment of RUF. The fistula was healed without recurrence at 1-year followup. Fibrin sealant injection has been used in anal and rectovaginal fistulas with an approximately 70% success rate [63, 64]. However, a prospective randomized trial for transsphincteric anal fistulae comparing fibrin glue treatment with seton treatment demonstrated that fibrin glue treatment had a significantly inferior probability of success [65]. Further successful cases using this procedure should be reported to confirm the excellent result.

Another method with cyanoacrylate glue was also reported. Bardari et al. [66] treated a patient with a neobladder-urethral fistula after radical cystoprostatectomy. They performed endoscopic application of cyanoacrylate glue, and the patient was disease free with no recurrence of RUF at a followup of 5 months. Bhandari et al. [67] also reported a successful case of a patient with RUF after RP. The patient was followed up 9 months after catheter removal without rectal leakage of urine. Although Bardari et al. [66] reported excellent results for this method of treatment of a prostate-perineal fistula after suprapubic prostatectomy and a neobladder-ileal fistula after radical cystoprostatectomy, further cases are necessary to validate those results. 

Thus these approaches using a sealant or glue are not yet mainstays of treatment for RUF.

## 7. Treatment for Recurrent RUF

There have been few reports of treatment for recurrence after RUF repair. Some infill, for example, an omental or gluteus muscle flap, fibrin glue, and so forth, can be considered. Alternatively, the coloanal sleeve anastomosis (Soave procedure) can be selected. The Soave technique was originally developed for treatment of Hircshsprung's disease [68]. Chirica et al. [58] reported its application for RUF treatment after RP with a 100% cure rate. The left colon is transected, and rectal mucosectomy is completed to the level of the RUF and via a perineal approach from the dentate line. After externally closing the urinary fistula (if possible), the stapled colon is delivered to the anus. Then the colon is transected at the level of the dentate line, and a coloanal anastomosis is performed. Although this procedure is more invasive, it may be the ultimate treatment option for complex RUF [58].

However, recurrent RUF is the most challenging problem. Kasraeian et al. [40] reported 3 patients who required multiple York-Mason procedures without a significant increase of intraoperative or postoperative morbidity. They also suggested that a second or third operation should be performed more than 5 months after the previous surgery. In general, repeated surgical failures can increase mortality and morbidity. Patients with nonrepairable RUF for whom prior attempts have failed should be considered for permanent urinary and fecal diversion. The options include cystoprostatectomy with an ileal conduit and proctectomy with continuation of the current fecal diversion.

## 8. Conclusions

Most of the techniques seem to provide high success rates (Table 2). The rectal flap method with the York-Mason approach and the gracilis muscle flap interposition are considered the most common procedures with high success rates and minimal morbidity. For radiated cases, gracilis muscle interposition may be preferred. However, since there has been no randomized clinical trial comparing the procedures because of the rarity of RUF, the best method is still unknown. The success of any surgical treatment assumes knowledge of all possible treatment methods. Recurrent or radiated RUF is the most challenging problem and sometimes requires permanent urinary and fecal diversion with proctectomy.

## Figures and Tables

**Table 1:** Posterior approaches to RUF.

	York-Mason	Kraske
Approach Position	Transsphincteric prone jackknife	Transsacral prone jackknife

	(1) Incision from the sacrococcygeal articulation to the anal verge	(1) Paracoccygeal incision 2–10 cm from the anal verge
	(2) Transection of entire sphincter complex in a layer-by-layer fashion	(2) Dissect down to and divide the anococcygeal ligament
Procedure	(3) Pairs of marking sutures at the mucocutaneous junction for resuture	(3) Resection of S4, S5, and coccyx
	(4) Midline division of the mucosa of the anus and the full thickness of the posterior rectal wall	(4) Midline division of the Waldeyer's fascia
	(5) Sleeve resection or proctotomy	(5) Sleeve resection or proctotomy

Complications	Fecal incontinence, fecal fistula	Fecal fistula

**Table 2:** Contemporary reports of RUF repair after RP.

Investigator	Year	Pts* (*n*)	Approach	Graft/infill	Closure technique	Success rate (%)
Pera et al. [36]	2008	5	York-Mason	—	Layer-to-layer	100
Crippa et al. [37]	2007	5	York-Mason	—	Layer-to-layer	100
Dafnis et al. [38]	2004	1	York-Mason	—	Layer-to-layer	100
Boushey et al. [39]	1998	2	York-Mason	—	Layer-to-layer	100
dal Moro et al. [10]	2006	4	York-Mason	—	Layer-to-layer	100
Renschler and Middleton [35]	2003	13	York-Mason	—	Layer-to-layer	100
Kasraeian et al. [40]	2009	12	Modified York-Mason	—	Layer-to-layer (only anterior rectal wall)	100
Spahn et al. [41]	2009	4	Transperineal	Buccal mucosa	Mucosal patch	75
Zmora et al. [42]	2006	2	Transperineal	Gracilis muscle flap	Layer-to-layer	100
Rivera et al. [43]	2007	6	Modified York-Mason or transperineal	— or gracilis muscle flap	Rectal flap or layer-to-layer	100
Ghoniem et al. [44]	2008	10	Transperineal	Gracilis muscle flap	Rectal flap	100
Ulrich et al. [45]	2009	4	Transperineal	Gracilis muscle flap	Layer-to-layer	100
Culkin and Ramsey [46]	2003	3	Transperineal	Deepithelialized scrotal flap	Y-V plasty	100
Quazza et al. [47]	2009	2	Transperineal	Omental flap mobilized laparoscopically	Layer-to-layer	100
Youseff et al. [48]	1999	2	Transperineal	Dartos pedicled flap	Layer-to-layer	100
Visser et al. [49]	2002	3	Transperineal	—	Rectal flap	100
Bochove-Overgaauw et al. [50]	2006	2	Transanal endoscopic	—	Layer-to-layer	50
Quinlan et al. [51]	2005	1	Transanal endoscopic	—	Layer-to-layer	100
Wilbert et al. [52]	1996	2	Transanal endoscopic	Fibrin glue	Layer-to-layer	100
Hata et al. [53]	2002	1	Transanal	—	Rectal flap	100
Noldus et al. [8]	1999	5	Transanal	—	Latzko	100
Joshi et al. [3]	2010	4	Transanal	—	Rectal flap	100
Sotelo et al. [54]	2005	1	Laparoscopic	Omental flap	Layer-to-layer	100
Sotelo et al. [55]	2008	1	Robotic	Omental flap	Layer-to-layer	100
Abdalla [56]	2009	1	Posterior sagittal pararectal with rectal mobilization	Gluteus muscle flap	Layer-to-layer	100
Castillo et al. [57]	2006	3	Anterior, transanal, transsphincteric, sagittal approach	—	Layer-to-layer	100
Chirica et al. [58]	2006	4	Intraperitoneal and perineal	Omental flap	Soave	100

*Patients who underwent radical prostatectomy.
